# A Time Study of Physicians’ Work in a German University Eye Hospital to Estimate Unit Costs

**DOI:** 10.1371/journal.pone.0121910

**Published:** 2015-03-24

**Authors:** Jan Wolff, Paul McCrone, Anita Patel, Gerd Auber, Thomas Reinhard

**Affiliations:** 1 Centre for the Economics of Mental and Physical Health, Institute of Psychiatry, Psychology & Neuroscience, King’s College London, London, United Kingdom; 2 Department of Management and Controlling, Medical Centre—University of Freiburg, Freiburg, Germany; 3 Eye Centre, Medical Centre—University of Freiburg, Freiburg, Germany; London School of Hygiene and Tropical Medicine, UNITED KINGDOM

## Abstract

**Background:**

Technical efficiency of hospital services is debated since performance has been heterogeneous. Staff time represents the main resource in patient care and its inappropriate allocation has been identified as a key factor of inefficiency. The aim of this study was to analyse the utilisation of physicians’ work time stratified by staff groups, tasks and places of work. A further aim was to use these data to estimate resource use per unit of output.

**Methods:**

A self-reporting work-sampling study was carried during 14-days at a University Eye Hospital. Staff costs of physicians per unit of output were calculated at the wards, the operating rooms and the outpatient unit.

**Results:**

Forty per cent of total work time was spent in contact with the patient. Thirty per cent was spent with documentation tasks. Time spent with documentation tasks declined monotonically with increasing seniority of staff. Unit costs were 56 € per patient day at the wards, 77 € and 20 € per intervention at the operating rooms for inpatients and outpatients, respectively, and 33 € per contact at the outpatient unit. Substantial differences in resources directly dedicated to the patient were found between these locations.

**Conclusion:**

The presented data provide unprecedented units costs in inpatient Ophthalmology. Future research should focus on analysing factors that influence differences in time allocation, such as types of patients, organisation of care processes and composition of staff.

## Introduction

Efficient allocation of resources in health care is under increasing scrutiny due to decreasing public budgets[1,2]. In particular, technical efficiency of hospital services is debated since performance of management has been heterogeneous [3,4]. Staff time represents the main resource in inpatient care and was estimated to account for about two thirds of total hospital costs [5]. Insufficient management of staff and their allocation of work time has been identified as a key factor of inefficiency in hospitals [6]. Of particular political interest has been the share of time physicians spent in direct contact with the patient and the time required for other obligations, such as paperwork [7].

Evaluating the efficiency of health care interventions requires quantification of resource consumption [8]. Two main approaches for the analysis of work time utilisation are time-and-motion studies and work-sampling. Time-and-motion studies require continuous observations of the study’s subject, often by external investigators following the subject throughout the working day [9]. They were applied to a variety of medical subjects, such as internal medicine [10], surgery [11], gynaecology [12], paediatrics [13] and psychiatry [14].

In contrast, work-sampling uses snap-shots of subject’s activities, usually at random points of time, in order to estimate total work time distribution [15]. Work-sampling was found to produce results of similar validity in comparison to time-and-motion studies [16] and its efficient design allows incorporating large numbers of staff [17].

The aim of this work-sampling study was to analyse the utilisation of physicians’ work time stratified by staff groups, tasks and places of work. A further aim was to use these data to estimate resource use per unit of output. To the best of our knowledge, the presented study is the first one of comparable scale in the field of Ophthalmology, where staff time allocation is of particular interest, due to its multifaceted services at the overlap of surgery and medical care.

## Methods

All physicians involved in clinical care at the Eye Centre of the Medical Centre—University of Freiburg were invited to participate in a 14-day study of their work time in October 2014. The Eye Centre has three wards with 34, 17, and 9 beds, respectively. It provides care for 5,500 inpatient episodes per year with a mean length of stay of 3.2 days and a case-mix index of 0.73. Furthermore, 22,000 surgical interventions are carried and 65,000 outpatient contacts are served per year. The top-five documented inpatient main diagnoses from the International Classification of Diseases (ICD-10) in 2013 were H40 Glaucoma (23%), H25 Senile cataract (15%), H33 Retinal detachments and breaks (10%), H02 Disorders of the eyelid (9%) and H35 Other retinal disorders (7%).

A handheld device specifically for the purpose of work-sampling studies was used, consisting of a pager and documentation forms. The pager gave random request during 30-minutes intervals, upon which participants documented their current place and type of activity. The study included each physician’s whole workday, starting and ending in the locker room with changing clothes. On-call duties were excluded, such as at the weekend and during the night. Four types of activities were surveyed. Direct care comprised all activities in contact with the patient, such as admissions and operating procedures. Indirect care comprised activities dedicated to one or more specific patients without their presence, such as medical reports and other documentations. General clinical time comprised activities not attributable to specific patients but required for the delivery of clinical care, such as staff scheduling and organisation. The category ‘other’ comprised all non-clinical activities, such as moving between units, research, teaching and breaks.

The surveyed places were three wards, a general unit of operating rooms (OR), a unit of OR for outpatient surgical interventions and a general outpatient unit. Research units and other places not dedicated to clinical care were surveyed in order to exclude time spent there from analysis of resource use.

Furthermore, participants documented their level of seniority. Assistants were medical graduates absolving a five-year specialist training in Ophthalmology, consultants were physicians who have completed their specialist training and seniors were appointed as directors of specific entities at the Centre.

The study was anonymous, its intention was described in advance and representatives of staff were involved early in the development of its design in order to reduce observer effects. Consent was given in advance and confirmed with handing in the documentations. Furthermore, the study and its methodology were approved by the works council of the University Medical Centre Freiburg and by the ethics committee of the Albert-Ludwigs-University of Freiburg.

Total number of patient days, surgical intervention and outpatient contacts were derived from the patient administration database. Estimated proportions were multiplied by total work time and the product was divided by the number of output units to calculate minutes of resource use per unit of output. Average personnel costs at the Eye Centre of the Medical Centre- University of Freiburg, including non-wage labour costs were used to monetarily value estimated staff time. Confidence intervals of work time proportions and associated hospital costs were calculated. Adjacent observations were not independently distributed but correlated by proximity, since the likelihood of an activity being observed has been influenced by the previously documented activity. Therefore, robust standard errors were calculated to adjust for autocorrelation between subsequent observations by clustering each working shift. Listwise deletion was used to address missing places or types of activities since the number of missing observations was very small (<1%) and negative effects on validity of results were unlikely [18].

## Results

A total of 6,419 observations were made of 39 participating physicians, representing 3,209 working hours. The response rate was 95%. Table 1 shows the distribution of work time among places and types of activities. Nineteen per cent of total work time was spent at the wards and 14% was spent at the ORs. The largest time-share was designated to the outpatient unit (39%). The remainder was spent at other places, such as laboratories, offices and meeting rooms.

**Table 1:** Distribution of total work time among types and places of physician activities.

	ward	or	outpat. or	outpatient unit	other	total
direct care	8,30%	8,07%	4,69%	18,49%	0,41%	39,96%
	(7,66%–9,01%)	(7,43%–8,76%)	(4,2%–5,24%)	(17,57%–19,47%)	(0,28%–0,59%)	(38,77%–41,17%)
indirect care	7,77%	1,00%	0,25%	16,87%	4,11%	30,00%
	(7,14%–8,46%)	(0,78%–1,27%)	(0,15%–0,41%)	(15,98%–17,81%)	(3,65%–4,63%)	(28,91%–31,13%)
general clinical	1,82%	0,05%	0,02%	1,28%	7,51%	10,67%
	(1,52%–2,18%)	(0,02%–0,14%)	(0%–0,11%)	(1,03%–1,58%)	(6,89%–8,18%)	(9,94%–11,45%)
other	0,86%	0,19%	0,03%	2,49%	15,80%	19,36%
	(0,66%–1,11%)	(0,11%–0,33%)	(0,01%–0,12%)	(2,14%–2,9%)	(14,92%–16,71%)	(18,42%–20,34%)
total	18,76%	9,30%	4,99%	39,13%	27,82%	100,00%
	(17,81%–19,73%)	(8,61%–10,04%)	(4,48%–5,55%)	(37,94%–40,32%)	(26,74%–28,93%)	

percentages: maximum-likelihood estimators, parentheses: 95% confidence interval, or: operation room, outpat.: outpatient.

Four fifth of total work time was dedicated to clinical care, evenly distributed among direct care (40%) and clinical activities without patient contact (41%), i.e. indirect care and general clinical time. The proportions of work time allocated among types of activities differed between places. While more than half of total work time at the ward was required for indirect care and general clinical time, almost 90% of time at ORs was spent in direct contact with the patient.

Table 2 shows the distribution of work time among places compared between assistant, consultant and senior physicians. Assistant physicians spent the main part of their time at the wards and the outpatient unit. Consultant and senior physicians were more likely to be located at the ORs and at other places, such as offices and meeting rooms.

**Table 2:** Distribution of work time among places stratified by staff groups.

	assistant	consultant	senior	unknown
	n = 3,572	n = 657	n = 1,898	n = 292
ward	27,63%	6,09%	8,06%	8,22%
	(26,19%–29,13%)	(4,5%–8,2%)	(6,92%–9,37%)	(5,57%–11,97%)
or	2,18%	13,85%	21,87%	4,45%
	(1,75%–2,72%)	(11,42%–16,71%)	(20,07%–23,78%)	(2,6%–7,52%)
outpatient or	2,41%	4,57%	9,48%	8,22%
	(1,95%–2,97%)	(3,21%–6,46%)	(8,24%–10,89%)	(5,57%–11,97%)
outpatient unit	49,66%	29,83%	20,55%	52,05%
	(48,03%–51,3%)	(26,44%–33,45%)	(18,79%–22,43%)	(46,31%–57,74%)
other	18,11%	45,66%	40,04%	27,05%
	(16,88%–19,4%)	(41,87%–49,49%)	(37,85%–42,26%)	(22,27%–32,45%)
total	100,00%	100,00%	100,00%	100,00%

n: number of observations, percentages: maximum-likelihood estimators, parentheses: 95% confidence interval.

or: operation room, unknown: missing documentation of seniority level.

Table 3 shows differences in work time allocation between types of activities. Assistants required the main share of their total work time for indirect care (40%), for example producing medical reports or other documentations. Time dedicated to indirect care declined monotonically with increasing level of seniority to 24% in consultant and 13% in senior physicians. Senior physicians dedicated the main share of their time to direct patient care (52%) and general clinical activities, such as staff scheduling and organisation (18%).

**Table 3:** Distribution of work time among activity types stratified by staff groups.

	assistant	consultant	senior	unknown
	n = 3,572	n = 657	n = 1,898	n = 292
direct care	34,69%	36,23%	51,69%	36,64%
	(33,14%–36,26%)	(32,63%–39,99%)	(49,44%–53,94%)	(31,3%–42,34%)
indirect care	39,53%	23,74%	13,07%	37,67%
	(37,94%–41,14%)	(20,64%–27,15%)	(11,62%–14,65%)	(32,28%–43,39%)
general clinical	7,03%	10,05%	18,12%	8,22%
	(6,23%–7,91%)	(7,97%–12,59%)	(16,45%–19,92%)	(5,57%–11,97%)
other	18,76%	29,98%	17,12%	17,47%
	(17,51%–20,07%)	(26,6%–33,6%)	(15,5%–18,89%)	(13,52%–22,27%)
total	100,00%	100,00%	100,00%	100,00%

n: number of observations, percentages: maximum-likelihood estimators, parentheses: 95% confidence interval,

unknown: missing documentation of seniority level.

**Fig 1:**
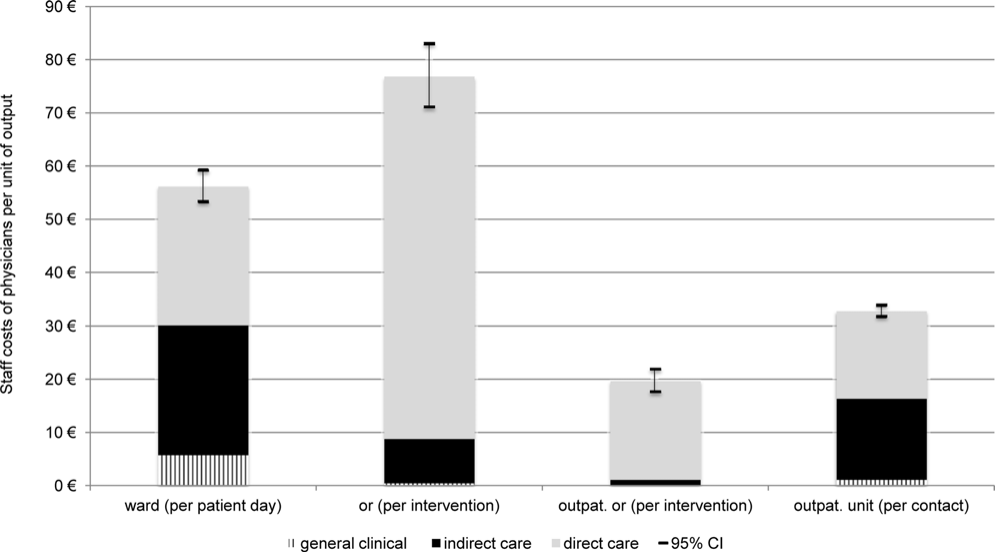
Monetarily valued staff time per unit of output. or: operation room, outpat.: outpatient.

Fig. 1 shows the monetarily valued use of staff time per unit of output. A patient day at one of the three wards represented staff costs of 56 € in total, thereof 24 € for indirect care (43%). Costs at the general OR represented 77 € per interventions, mainly dedicated to direct patient care (68 €). Total costs per contact at the outpatient unit were two thirds higher than total costs per intervention at the outpatient OR with 33 € in comparison to 20 €. However, cost for direct care at the outpatient OR (19 €) were higher than costs for direct care at the outpatient unit (17 €), due to high shares of time in direct contact with the patient at the outpatient OR.

## Discussion

The aim of this study was to analyse the utilisation of physicians’ work time. A further aim was to use these data to estimate resource use per unit of output. Forty per cent of total work time was spent in contact with the patient. Thirty per cent was spent with documentation tasks. Time spent with documentation tasks declined monotonically with increasing seniority of staff. Costs per unit of output were presented and substantial differences in resources directly dedicated to the patient were found between wards, ORs and the outpatient department.

The share of time in direct contact with the patient (40%) appears high in comparison with previously conducted studies. Westbrook et al [19] analysed physicians’ work time distribution at respiratory, renal/vascular and geriatric medical wards and found physicians to spent between 17% and 28% of total working in direct contact with the patient. Furthermore, O’Leary et al [10], Weigl et al [11] and Kloss et [12] found 18%, 34% and 30% of total working time in direct contact with the patient in internal medicine, surgery and gynaecology, respectively.

The results of work time analyses might not be directly transferable between health care systems, since organisational patterns, hierarchy levels and average income might be different. Furthermore, methodological aspects might have influenced results. The abovementioned studies restricted their analysis to junior physicians. In contrast, the presented study included participants from all ranks of hierarchy and found the less experienced staff to require more time for indirect care. These finding was supported by Westbrook et al in 2008 [20], who also found time required for indirect care to decrease monotonically with increasing seniority from 19% in interns to 17% in residents and 14% in registrars. Furthermore, Gabow et al [13] found that residents in their second year required only about half the time for charting that was required by first years residents. A possible explanation for more time required for indirect care by junior staff is more efficient fulfilment of required tasks by more experienced staff. However, it might also be possible that unpopular tasks have been passed down the medical hierarchy.

A strength of the presented study has been the large sample size. This scale would have been prohibitively costly with other methods, such as time-and-motion studies. However, there are two potential caveats to all time studies. First, the study period might not have been representative of regular clinical conditions since admitted patients might have been different or other factors could have altered usual circumstances. This caveat was addressed by ex ante choosing a study period that was expected to be mainly representative of the annual average, for instance without unusually high absence due to factors such as major conferences. Furthermore, ex post analyses showed no substantial differences in output figures (not shown in results) and clinical staff reported no unusual events.

Second, study subjects might have altered their behaviour as a mere consequence of their awareness of being investigated [21]. This caveat was addressed with methodological aspects of the study design. In particular, Levitt and List [22] presented evidence that two aspects should have reduced distorting influence of observer effects, meaning strict anonymisation of the study subjects to the investigator and the absence of an external observer following the participants. Nevertheless, it has been impossible to completely rule out observer effects.

The study presented a detailed description of the allocation of a main resource in inpatient care, namely the time of physicians, and resulting costs per unit of output were presented. These data provided a fundament for analysing the efficiency of the delivery of care. They should be used for continuous controlling of achieved efficiency by longitudinal surveillance of performance developments and the identification of areas of potential improvement. Future research should include other aspects of resource use, such as nursing and material costs. Furthermore, more focus should be given to the analysis of factors that influence differences in time allocation, meaning types of patients, organisation of care processes and composition of staff.
